# Acupuncture on GB34 for immediate analgesia and regulating pain-related anxiety for patients with biliary colic: a protocol of randomized controlled trial

**DOI:** 10.1186/s12906-023-04030-8

**Published:** 2023-07-07

**Authors:** YuanFang Zhou, YuQuan Shen, XiangYin Ye, DongMei He, Ning Sun, Yong Zhang, YaFei Zhang, Chao Long, ShanBin Ding, LiPing Deng, Yi Deng, FanRong Liang, XianTian Gong, RuiRui Sun

**Affiliations:** 1 Acupuncture and Tuina School/The 3rdTeaching Hospital, Chengdu University of Traditional Chinese Medicine/ Clinical Research Center for Acupuncture and Moxibustion in Sichuan Province, 37 Shierqiao Road, Chengdu, Sichuan China; 2 Department of Rehabilitation Medicine, The First People’s Hospital of Longquanyi District, Chengdu, 610100 Sichuan China; 3 ChongQing JiangJin District Hospital of Chinese Medicine, ChongQing, China; 4 Emergency Department, The First People’s Hospital of Longquanyi District, Chengdu, 610100 Sichuan China; 5 Rehabilitation Medicine Center and Institute of Rehabilitation Medicine, West China Hospital, Sichuan University, Chengdu, 610041 Sichuan China; 6 Meishan Hospital of Traditional Chinese Medicine, 14 Suci Road, Dongpo District, Meishan, 620010 Sichuan China; 7 Acupuncture and Brain Research Center, Chengdu University of Traditional Chinese Medicine, Chengdu, China

**Keywords:** Biliary colic, Acupuncture, Randomized controlled trial, Protocol

## Abstract

**Background:**

Biliary colic (BC) is a frequent hepatobiliary disorder encountered in emergency departments. Acupuncture may be effective as an alternative and complementary medicine for BC. Nonetheless, rigorous trials investigating its efficacy are lacking. Therefore, the aim of this study protocol is to determine whether acupuncture provides immediate relief of pain and associated symptoms in BC patients.

**Method:**

Eighty-six participants who aged from 18 to 60 years with BC will be recruited in the First People's Hospital of Longquanyi District, Chengdu (West China Longquan Hospital Sichuan University). All participants will be allocated into two treatment groups including acupuncture group and sham acupuncture group using a 1:1 ratio. Each group will only receive a single 30-min needle treatment while waiting for their test results after completing the routine examination for BC. The primary outcome of the study is to assess the change in pain intensity after the 30-min acupuncture treatment. The secondary outcomes of the study include the change in pain intensity at various time points, the degree of gastrointestinal symptoms at different time points, the level of anxiety experienced during pain episodes at different time points, the score of Pain Anxiety Symptoms Scale-20 (PASS-20), the score of Fear of Pain Questionnaire-III (FPQ-III), and the score of Pain Catastrophizing Scale (PCS), among others.

**Discussion:**

The results of this research will provide substantial evidence regarding the efficacy of acupuncture in alleviating symptoms associated with BC.

**Trial registration:**

ClinicalTrials.gov, ChiCTR2300070661. Registered on 19 April 2023.

**Supplementary Information:**

The online version contains supplementary material available at 10.1186/s12906-023-04030-8.

## Background

Biliary Colic (BC) is a symptom of an acute attack of gallstones. The prevalence of gallstones has been displayed to be 10%-20% patients in worldwide. Furthermore, more than 20% of patients with gallstones will develop BC symptom [1]. It is estimated that complications occur in approximately 1%–3% of patients with symptomatic gallstones annually [2]. BC is characterized by persistent pain in the upper part of the abdomen, back, or right shoulder, as well as nausea and vomiting, which are caused by the contraction of the gallbladder pushing bile across the blockage [3].

The affective component of pain includes feelings of distress, sadness, anxiety, and depression in response to noxious stimuli [4]. Depression and anxiety have been linked to prolonged duration of acute pain [5]. Likewise, BC, being an acute pain, may be clinically accompanied by emotional symptoms such as anxiety and depression. Studies on pain-induced anxiety have demonstrated that acute pain intensity is positively correlated with anxiety status [6, 7]. Literature on pain-induced depression has suggested that acute pain may contribute to depressive symptoms following surgery [8].

An evidence-based clinical practice guideline for the remission of BC has recommended non-steroidal anti-inflammatory drugs (such as diclofenac and indomethacin), spasmolytics (such as butylscopolamine), opioids (such as buprenorphine), and cholecystectomy (within 24 h after the diagnosis of biliary colic) [9–11]. However, pharmacological treatments may have side effects, including serious gastrointestinal complications, cardiovascular events, hypersensitivity, itch, nausea, and vomiting, among others [12–17]. Additionally, cholecystectomy may be accompanied with post cholecystectomy syndrome [18, 19]. Thus, complementary alternative therapy is considered essential to improve BC symptoms.

Acupuncture, as part of traditional Chinese medicine, potentially relieves the pain-related disorders [20–22]. Some clinical studies have proved that acupuncture can alleviate pain-related symptoms of BC [23, 24]. Moreover, our team found that acupuncture for BC is effective through the imaging mechanism of acupuncture at GB34 in the early stage. However, the research has mainly focused on the mechanism of pain, and the sample size was relatively small, despite patients with BC often experiencing emotional problems [25, 26]. Therefore, thus far, it is unclear whether acupuncture has analgesic effects on BC owing to the methodological limitations in control groups, randomization, and blinding, among other factors.

Hence, a randomized, sham-controlled trial will be conducted to assess the potential of acupuncture in improving pain and related symptoms of BC.

## Methods/design

### Study design

**Fig. 1:**
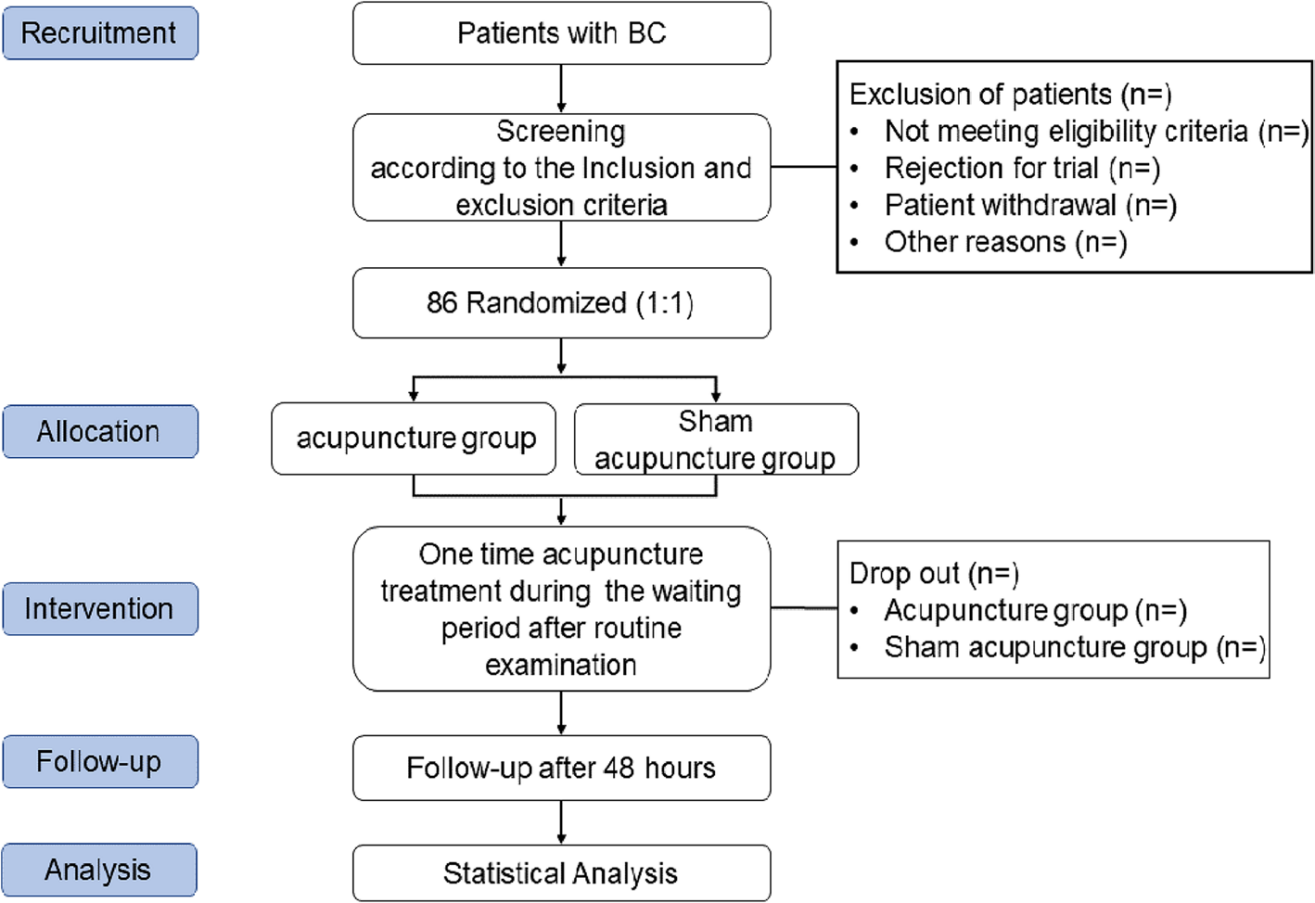
Trial flow chart

The single center, sham-controlled, blinded, randomized trial of acupuncture treatment for BC will be executed in the First People's Hospital of Longquanyi District of Chengdu (West China Longquan Hospital Sichuan University) from May 2023 to July 2024. The protocol of trial has been approved by the Affiliated Hospital of Chengdu University of Traditional Chinese Medicine (No. 2023KL-008). And it has been registered with an identifier (ChiCTR2300070661) at ClinicalTrials.gov (https://www.chictr.org.cn/). The trial will be performed in accordance with the Standard Protocol Items: Recommendations for Interventional Trials (SPIRIT) checklist [27]. Moreover, informed consent will be obtained from all patients prior to beginning the trial. All BC patients will accept acupuncture treatment while patients are waiting for the test results after completing routine examinations. If patients with BC continue to experience pain after acupuncture treatment, conventional treatment will be administered, and the pain relief time will be recorded. The study flow chart is shown in Fig. 1, and the study time schedule is presented in Table 1.

**Table 1:**
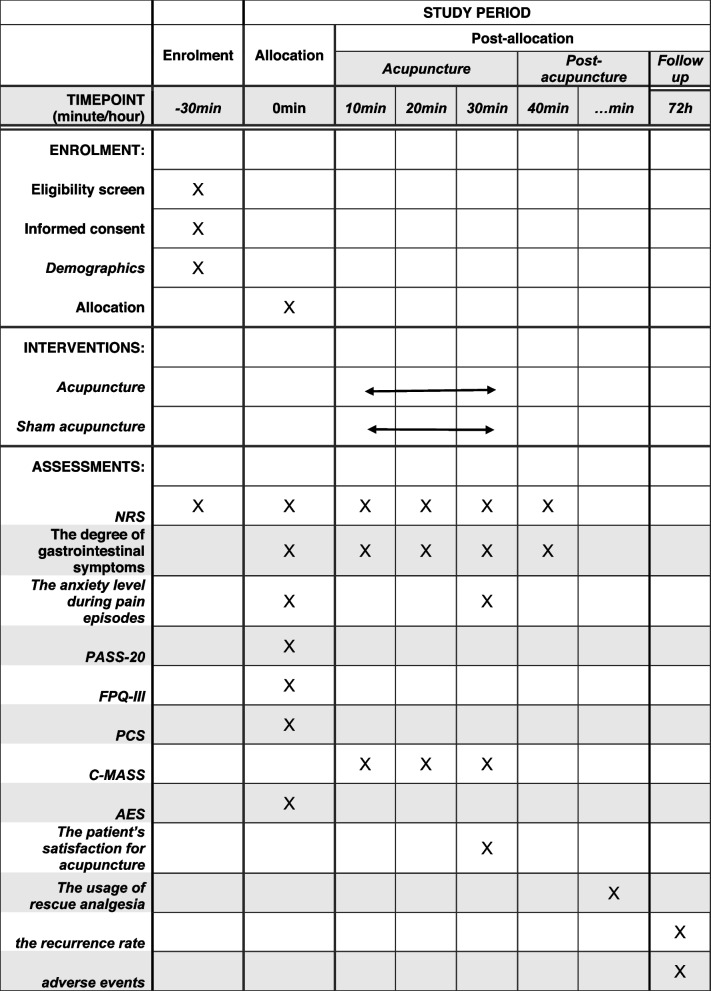
Trial processes chart

The use of rescue analgesia will be recorded 10 min after the completion of acupuncture, As the specific time for each subject to use the analgesic may vary, "…" will be used to indicate the time

### Participants

Eighty-six patients with BC will be recruited from the emergency department of the First People's Hospital of Longquanyi District of Chengdu (West China Longquan Hospital Sichuan University). All patients will be screened by Specialists and acupuncturists according to the diagnostic criteria from the Recommendations for EASL clinical practice guidelines on the prevention, diagnosis and therapy of gallstones (2016) [9].

### Inclusion criteria

Participants who meet the following inclusion criteria will be eligible for the study: (i) those who meet the diagnostic criteria; (ii) mild tenderness in the right upper quadrant, negative or positive Murphy's sign; (iii) a numerical rating scale (NRS) score of ≥ 3 points; (iv) BC patients aged between 18 to 60 years, without any gender restrictions; (v) gallbladder stones demonstrated by radiology or ultrasonography; (vi) no history of acupuncture treatment within the past month; (vii) not participating in any other clinical studies; and (viii) willing to voluntarily sign the informed consent form.

### Exclusion criteria

Participants who meet the following exclusion criteria will be excluded from the study: (i) a history of jaundice; (ii) presence of bile duct stones, acute cholecystitis, acute suppurative cholecystitis, gangrenous cholecystitis, incarcerated cholecystitis, gallbladder perforation with diffuse peritonitis, or BC complicated with acute pancreatitis; (iii) administration of analgesics such as antispasmodic, non-steroidal anti-inflammatory drugs, or choleretic before acupuncture; (iv) presence of serious complications or primary diseases involving the brain, cardiovascular, liver, kidney, endocrine, or hematopoietic systems; (v) presence of serious digestive system diseases, such as peptic ulcer, upper gastrointestinal bleeding, gastric tumor, Crohn's disease, irritable bowel syndrome, etc.; and (vi) participation in other clinical trials within the last three months.

### Recruitment procedures

BC patients in emergency department of the First People's Hospital of Longquanyi District (West China Longquan Hospital Sichuan University) will be diagnosed by emergency physician. Simultaneously, researchers will be responsible for the screening and presenting the inclusion criterion, the operational and allocated details of intervention (acupuncture group and sham acupuncture group), and the potential risks of this trial. All subjects will have the right to withdraw from the trial at any time. Additionally, all subjects will be required to sign an informed consent form before the trial commences.

### Randomization and allocation

Baseline evaluation will be conducted for eligible subjects. The random sequence will be generated using SAS® 9.4 software (SAS Institute Inc., North Carolina, USA). The random sequence will be recorded on sealed and opaque envelopes, with the random numbers of the two groups inside. Moreover, these envelopes will be opened sequentially by a researcher assistant who is not involved in the data collection or acupuncture treatment. In addition, the researcher assistant will randomly assign the subjects to the acupuncture group and the sham acupuncture group with ratio of 1:1.

### Blinding

The researcher assistants, outcome assessors, statisticians and subjects will be blinded to the group assignment. All subjects will receive acupuncture treatment lying on the bed in a quiet clinic. The control group will use a non-penetrating sham apparatus, similar in appearance to acupuncture needles, to achieve blinding. Furthermore, all subjects will be asked about their experience after the acupuncture session to assess whether they have been successfully blinded.

### Interventions

#### Acupuncture group

**Fig. 2:**
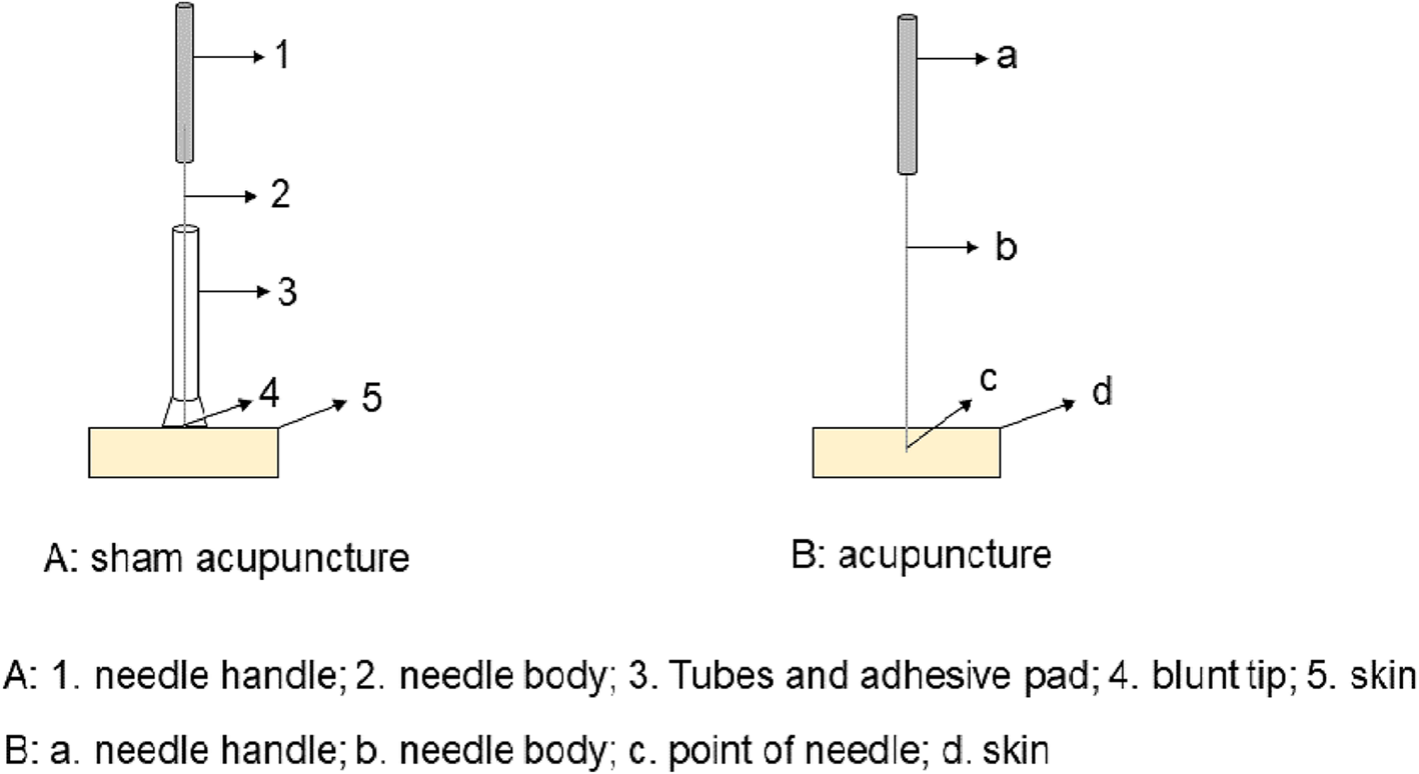
The diagram of acupuncture and non-penetrating sham apparatus

Yanglingquan (GB34) is acupoint of the gallbladder meridian of foot-shaoyang (GB), which is frequently used for Biliary disease [28, 29]. Therefore, right GB34 will be chosen as the acupuncture point to treat BC. Acupuncture treatment will be performed by acupuncturists with more than 3 years of clinical experience. The acupuncturist will disinfect the skin around the right GB34 with a 70% isopropyl alcohol swab when the subjects lie on the treatment bed in a supine position. Disposable sterile acupuncture needles (0.25 mm in diameter and 40 mm in length, Hwatuo, Suzhou, China) will then be inserted in the right GB34 (Fig. 2B). The needle will be lifted and thrust approximately 30–50 mm and twisted and rotated approximately 90° to 180° angle for 60–90 times/min to achieve deqi sensation, including soreness, numbness, heaviness, fullness, and aching. The deqi sensation will be evaluated using the Chinese version of the modified Massachusetts General Hospital Acupuncture Sensation Scale (C-MASS). All subjects will receive one 30-min acupuncture treatment during their waiting time after routine examination, and the manipulations will be executed two times, once every 10 min, for 60 s each time.

#### Sham acupuncture group

The right non-acupuncture point located at the lateral edge of the tibia, approximately 1–2 cm away from Zusanli (ST36), will be selected as the sham point [30]. A non-penetrating sham apparatus with a blunt tip, specifically the Park sham device, will be used to lightly touch the skin at the sham point [31] (Fig. 2A). The procedures and manipulations in the non-acupuncture group will be identical to those in the acupuncture group. Subjects will receive treatment alone in a single room to prevent any interference from family members or other individuals.

### Outcome measures

#### Primary outcome measures

The primary endpoint is the change of pain intensity from baseline to 30 min after acupuncture by evaluating the NRS. The NRS is a 10-point scale, with zero representing "no pain" and 10 representing "worst possible pain". The pain intensity of BC subjects will be presented by marking a “√” on the line of NRS.

#### Secondary outcome measures

The secondary end points will be evaluated as following:

i) The pain intensity will be assessed the change of NRS score from baseline to the 10 min, 20 min and 40 min after needle manipulation.
ii) The degree of gastrointestinal symptoms, such as nausea, vomiting and pantothenic acid, will be evaluated for change from baseline by the visual analogue scale (VAS) at 10 min, 20 min, 30 min and 40 min after needle manipulation [32].
iii) The level of anxiety during episodes of pain will be measured by changes in the VAS at 10 min, 20 min and 30 min after needle manipulation, compared to baseline [33]. The VAS will be used to assess the anxiety level of patients with BC during pain attacks.
iv) The pain-related emotional responses, such as anxiety and fear, will be assessed using the Pain Anxiety Symptoms Scale-20 (PASS-20), the Fear of Pain Questionnaire-III (FPQ-III), and the Pain Catastrophizing Scale (PCS) at baseline [34–37]. The PASS-20 will be used to evaluate whether patients with biliary colic experience anxiety during the non-pain periods.
v) The patients' anticipated responses to acupuncture will be evaluated using the Acupuncture Expectancy Scale (AES) at baseline [38].
vi) The patient’s satisfaction for acupuncture will be monitored by the 5-point Likert scale at 30 min after needle manipulation [39].
vii) The evaluation of needle sensation will be conducted using the Chinese version of the modified Massachusetts General Hospital Acupuncture Sensation Scale (C-MASS) at 10 min, 20 min and 30 min after needle manipulation [40].
viii) The use of rescue analgesia will be recorded 10 min after the completion of acupuncture. The recurrence rate of BC and any adverse events occurring within 72 h after needle manipulation will be obtained through a telephone follow-up.

### Safety assessment

The information of adverse events (AEs) related to interventions including infection, bloody swelling, stabbing pain, broken needle, hemorrhage, palpitation, syncope, headache will be monitored and recorded in the case report forms (CRF) by a research assistant. Serious AEs will be handled by emergency medicine physicians or acupuncturists. Moreover, serious AEs will be reported to the Ethics Committee of Hospital of Chengdu University of Traditional Chinese medicine.

### Data collection and quality control

The data will be collected alone on CRF by the same evaluator and will be imported into the computer respectively by two research assistants who are not involved in the trial to avoid bias in the results. All personnel involved in the study will be required to complete professional training to ensure the quality of the trial. This training will cover details of the trial, including the introduction of inclusion and exclusion criteria, completion of CRF, acupuncture procedures, and data entry methods.

### Sample size calculation

The primary outcome of this study is the mean difference in NRS scores for BC between the baseline and the endpoint. Sample size calculations will be based on a previous neuroimaging study of acupuncture in BC [41], which reported mean differences in NRS scores of -1.74 ± 1.543 for the acupuncture group and -0.66 ± 0.986 for the sham acupuncture group. Accordingly, a total of 76 patients will be calculated using a 2-sided test with a 5% significance level and 90% power. To account for a 10% dropout rate, this trial will ultimately enroll 86 patients.

### Statistical analysis

The independent samples t-test or Wilcoxon rank-sum test will be used to analyze continuous variables in the demographic characteristics of the two groups, based on their normality. Categorical variables in the demographic characteristics will be analyzed using chi-squared or Fisher's exact tests. If there is a significant difference in demographic characteristics such as age, gender, and family history, they will be included as covariates in the efficacy analysis. The analysis will include all randomized BC patients based on the intention-to-treat (ITT) principle. Subgroup and sensitivity analyses may also be performed.

The change of pain intensity from baseline to 30 min after acupuncture will be analyzed by using analysis of covariance with acupuncture group and sham acupuncture treatment as factors and baseline pain intensity as a covariate. Repeated measures ANOVA will be used to assess the difference in the secondary outcomes at different time points. The degree of patients’ expectations and satisfaction with acupuncture will be evaluated using linear regression.

## Discussion

BC is one of the 64 diseases that are suitable for acupuncture treatment, as recommended by the World Health Organization [42]. The studies of acupuncture treatment for BC have reported that acupuncture has play therapeutic effect on BC [43–46]. However, the quality of evidence supporting the use of acupuncture for BC is limited due to methodological limitations. Furthermore, some studies have shown that sham acupuncture, such as needle insertion into wrong or non-points, or superficial needling at non-points, may produce efficacious results [47, 48]. Therefore, it is necessary to use non-penetrating needling as the control group to produce a smaller, nonspecific effect [49].

Several clinical studies have proposed that acupuncture on GB34 could significantly improve the symptoms of patients with BC [50, 51]. Furthermore, GB34 is located on the lower limbs of the patient, making it difficult for them to observe the treatment procedure and needle site while in a supine position. Therefore, the selection of acupoints is likely to increase the likelihood of patient-blinding.

In this trial, there are several limitations that need to be acknowledged. Firstly, blinding of acupuncturists is not possible due to the unique nature of acupuncture procedures. Secondly, the small sample size may lead to inconclusive results. Finally, there is a potential for a relatively high dropout rate in the sham acupuncture group.

To summarize, this trial aims to investigate the efficacy and safety of acupuncture in relieving pain and pain-related symptoms in patients with BC by controlling the quality of acupuncturist’s operation and implementing assignment blinding. The results of this trial are expected to provide an alternative treatment option for patients with BC and contribute to the development of guideline recommendations.

### Trial status

The trial is currently recruiting participants. The first subject has not been recruited so far and the investigators are still collecting.

## Supplementary Information

**Additional file 1.**

## Data Availability

Not applicable.

## Abbreviations

BC: Biliary colic
SPIRIT: Standard Protocol Items: Recommendations for Interventional Trials
GB34: Yanglingquan
GB: Gallbladder meridian
C-MASS: Chinese version of the modified Massachusetts General Hospital Acupuncture Sensation Scale
ST36: Zusanli
NRS: Numerical rating scale
VAS: Visual analogue scale
PASS-20: Pain Anxiety Symptoms Scale-20
FPQ-III: Fear of Pain Questionnaire-III
PCS: Pain Catastrophizing Scale
AES: Acupuncture Expectancy Scale
AEs: Adverse events
CRF: Case report forms
ITT: Intention-to-treat
